# How does the use of quantified gap-balancing affect component positioning and limb alignment in robotic total knee arthroplasty using functional alignment philosophy? A comparison of two robotic platforms

**DOI:** 10.1007/s00264-022-05681-x

**Published:** 2023-02-06

**Authors:** Geoffrey T. Murphy, Jobe Shatrov, Julian Duong, Brett A Fritsch

**Affiliations:** 1 Sydney Orthopaedic Research Institute, Level 2, 500 Pacific Highway, St Leonards, St. Leonards, Sydney, 2065 Australia; 2 University of New South Wales, Sydney, Australia; 3 Sydney University, Sydney, Australia

**Keywords:** Robotically-assisted total knee arthroplasty, MAKO, OMNIbot, Measured resection, Gap-balancing, robotics

## Abstract

**Purpose:**

This study aimed to compare the effect of an image-based (MAKO) system using a gap-balancing technique with an imageless (OMNIbot) robotic tool utilising a femur-first measured resection technique.

**Methods:**

A retrospective cohort study was performed on patients undergoing primary TKA with a functional alignment philosophy performed by a single surgeon using either the MAKO or OMNIbot robotic systems. In all cases, the surgeon’s goal was to create a balanced knee and correct sagittal deformity (eliminate any fixed flexion deformity). Intra-operative data and patient-reported outcomes (PROMS) were compared.

**Results:**

A total of 207 MAKO TKA and 298 OMNIbot TKAs were analysed. MAKO TKA patients were younger (67 vs 69, *p*=0.002) than OMNIbot patients. There were no other demographic or pre-operative alignment differences. Regarding implant positioning, in MAKO TKAs the femoral component was more externally rotated in relation to the posterior condylar axis (2.3° vs 0.1°, *p*<0.001), had less valgus femoral cuts (1.6° vs 2.7° valgus, p<0.001) and more varus tibial cuts (2.4° vs 1.9° varus, *p*<0.001), and had more bone resected compared to OMNIbot TKAs. OMNIbot cases were more likely to require tibial re-cuts than MAKO (15% vs 2%, *p*<0.001). There were no differences in femur recut rates, soft tissue releases, or rate of achieving target coronal and sagittal leg alignment between robotic systems. A subgroup analysis of 100 MAKO and 100 OMNIbot propensity-matched TKAs with 12-month follow-up showed no significant difference in OKS (42 vs 43, *p*=0.7) or OKS PASS scores (83% vs 91%, *p*=0.1). MAKO TKAs reported significantly better symptoms according to their KOOS symptoms score than patients that had OMNIbot TKAs (87 vs 82, *p*=0.02) with a higher proportion of KOOS PASS rates, at a slightly longer follow-up time (20 months vs 14 months, *p*<0.001). There were no other differences in PROMS.

**Conclusion:**

A gap-balanced technique with an image-based robotic system (MAKO) results in different implant positioning and bone resection and reduces tibial recuts compared to a femur-first measured resection technique with an imageless robotic system (OMNIbot). Both systems achieve equal coronal and sagittal deformity correction and good patient outcomes at short-term follow-ups irrespective of these differences.

## Introduction

Successful total knee arthroplasty (TKA) requires precise implant positioning and alignment [1]. It has been observed that a significant proportion of patients experience ongoing symptoms following TKA, with rates up to 33–54% being reported [2]. Furthermore, disturbed gait kinematics and reduced range of motion are common [3]. These findings have led to a vigorous pursuit of tools and techniques to improve the accuracy and consistency of implant positioning, along with more patient-specific alignment strategies with the aim of improving clinical outcomes.

Evidence is emerging that tibio-femoral compartmental balancing affects pain and kinematics up to two years after TKA [4–8]. What defines a ‘balanced’ knee, and what thresholds are used to set boundaries on laxity remains the focus of much ongoing debate and research. A commonly utilized approach is one that aims for equal laxity in the medial and lateral compartments in full extension, with a slight lateral laxity being acceptable in flexion. This definition is based on the observation that the medial compartment is constrained by the rigid medial soft tissue structures of the knee (superficial and deep medial collateral ligament complex) and is relatively immobile, compared to the lateral compartment that shows a larger amount of femoral rollback and is more lax, particularly in flexion [9–16], similar to that seen in the native knee, and is supported by published literature [17].

There are multiple ways to achieve this balance; cut the bones perpendicular to the mechanical axis in the coronal plane, rotate the femur parallel to the transepicondylar axis in the axial plane, and match the tibial slope, and then sequentially release ligaments to overcome any residual imbalance (mechanical alignment philosophy). An alternate approach is to position the implants with the aim to recreate the anatomy of the native knee, making tibiofemoral resections that aim to recreate the pre-diseased knee with minimal soft tissue releases and accepting that patients native soft tissue laxity (kinematic alignment (KA) philosophy) [18]. A more recent technique has been described where the surgeon starts with a plan based on KA principles, but implant position is adjusted according to the patients’ soft tissue envelope to keep within defined limits and to also achieve tibiofemoral compartment balancing (functional alignment) [19]. In these cases, there are again many ways to reach the final implant position, with 2 common strategies being via a measured resection (bone cut depth to match implant thickness) or gap-balancing (alter bone cut depth based on the soft tissue laxity).

Measured resection involves positioning the implants to recreate the patients’ starting joint lines in all planes, accounting for the amount of chondral and/or bone loss compared to the healthy state. The distal femoral cuts in this method are planned parallel to the chondral lateral distal femoral angle (LDFA) the posterior femoral cuts parallel to the chondral posterior condylar axis (PCA) and the tibia parallel to the chondral medial proximal tibial angle (MPTA). Primary gap balancing involves selecting a starting point for implant position and then measuring the gaps that result and adjusting implant position to balance them. With traditional manual instrument tools, this involves a subjective assessment of the resultant balance by the surgeon’s feel. With navigation assistance, there was an improvement in being able to infer gaps from alignment curves. This has been advanced further with some robotic systems such as the MAKO that are able to predict gaps in a virtual environment prior to actual bone cutting, and then measure actual resultant gaps during surgery, and adjust implant at either step as required [20]. Consideration of gap targets means the wide natural variation of soft tissue laxity that exists between patients [16, 21, 22] can be incorporated into the positioning of the implant in all three planes using objective, reproducible and definable targets.

Multiple proprietary robotic platforms have been developed for TKA in the last decade, and the capabilities vary significantly between systems. The technique a surgeon decides to use will be influenced by the capabilities of the tool available to the surgeon for the execution of the surgery. Image-based systems (such as MAKO) re-create a 3D virtual model for pre-operative and intra-operative planning. This enhances the surgeon’s ability to anticipate issues that may rise from variations in patient anatomy and allows for pre-operative fine-tuning of implant positioning [23]. However, the cost and radiation exposure are disadvantages [24], as are the size and low transportability of the machine itself. Imageless systems such as OMNIbot reduce radiation exposure and do not incur the cost of pre-operative imaging, but subsequently rely on the operator’s accuracy to correctly register landmarks[25]. Anatomical mapping in cases of large deformity, bone loss, or post-trauma may be prone to registration error [26], and there is not an option for virtual gap balancing allowing pre-cut adjustment based on gap data in a femur-first approach. Effectively both systems allow for “gap balancing”, but in different ways and at different parts of the surgical workflow. The MAKO allows for it in a virtual environment with quantified virtual extension and flexion gaps and true 6-degree of freedom adjustment of both femoral and tibial components being possible because it’s done prior to bone cutting, whereas the OMNIbot is gap balanced by the surgeon after initial bone cuts (tibial first) with the surgeon calculating balance by subjective feel combined with inferences from alignment curves, and the adjustments being reactionary to the cuts already made resulting in some limitation in what options are available.

There is currently no literature comparing the resultant effect of these different robotic systems on implant position or outcomes. The aim of this study was to compare the effect of using an image-based (MAKO) robotic system with a complete virtual gap balancing technique feature compared to an imageless (OMNIbot) robotic tool with femur-first measured resection technique and alignment inferred gap quantification, on implant positioning and limb alignment in patients undergoing primary TKA. The hypothesis was that the image-based system would lead to less bone resection and a higher percentage of implants within the surgeon’s aimed implant range.

## Methods and materials

This study was performed in line with the principles of the Declaration of Helsinki. Approval was granted by the Northern Sydney Local Health District Human Research Ethics Committee (2019/ETH08340).

This was a retrospective review of prospectively collected data of consecutive patients undergoing primary TKA performed by a single high-volume arthroplasty surgeon at two institutions. All cases were undertaken between June 2018 to November 2021 using either the MAKO or OMNIbot robotic systems. This date was chosen to nullify the effect of the initial learning curve associated with using the MAKO system [27, 28].

Patients were included if they underwent a robotically assisted TKA using either the MAKO or OMNIbot robotic systems. Patients were excluded in cases of revision surgery, or if they received TKA by an alignment philosophy other than functional alignment. Patients received the Omni Apex (Global Orthopaedics, Australia) cruciate retaining implant or the MAKO Stryker Triathlon (Stryker, Kalamazoo, MI) cruciate retaining implant based on the robotic system utilised.

### Surgery

All surgery was performed via a medial parapatellar approach and functional alignment philosophy [29]. The patella was resurfaced in all cases, all osteophytes were removed prior to the initial balance assessment, and all components were cemented.

### MAKO TKA

For TKA performed with the MAKO, a virtual measured resection implant starting position with adjustments to the femur and tibia positions in the virtual environment *prior* to any bony cuts being made, based on *quantified virtual* gaps provided by the software. Both femur and tibia can be easily adjusted to achieve the surgeon’s desired implant position and balance concurrently.

### OMNIbot TKA

For TKA performed with OMNIbot, initial resections of femur and tibia were performed using measured resection principals with any further adjustments made *after* these initial bone cuts by recutting the tibia as needed to achieve balance goals using surgeon-based feel and navigation curves to guide the tibial recut (effectively gap balancing the tibia of a fixed measured-resection femur).

Once trial components were inserted, the navigation was used to measure the range of motion and laxity and (in MAKO cases) gaps. The goal in the sagittal plane was to achieve full extension under gravity, (defined as 2° flexion to 2° hyperextension holding the foot by the heel and lifting the leg). Where full extension was not achieved, a posterior capsular release was performed. If the capsular release was insufficient to achieve full extension with the smallest polyethylene insert, a re-resection of the distal femur would be performed. Any releases or extra resections were recorded. Where the knee was hyperextending, thicker polyethylene inserts were used until this was corrected. Tightness in flexion was assessed intra-operatively (surgeon feel in the OMNIbot, measured gaps with the MAKO) and addressed by changing the tibial slope angle.

### Coronal balancing

Functional alignment was defined as follows:

I. Femoral component: within 6° valgus and 1° varus
II. Tibia component: within 5° varus and 1° valgus
III. Final limb alignment: hip-knee-ankle (HKA) angle within 5° varus and 3° valgus

Coronal plane balancing was assessed throughout a range of motion, with the aim of achieving equal gaps (within 1mm) medial and lateral in extension (0°). In 90° flexion, more lateral laxity was tolerated. Balancing was considered achieved if the medial and lateral gap were equal (within 1mm) in extension, the medial gap was equal in extension and flexion (within 1mm), and the lateral flexion gap was within 0–3mm of the medial flexion gap.

**Fig. 1:**
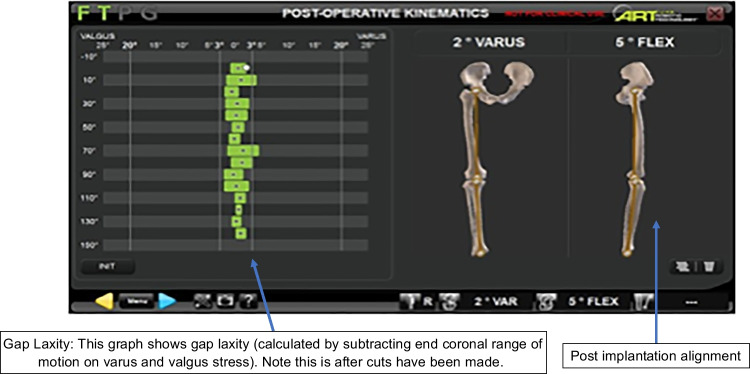
OMNIbot gap assessment

**Fig. 2:**
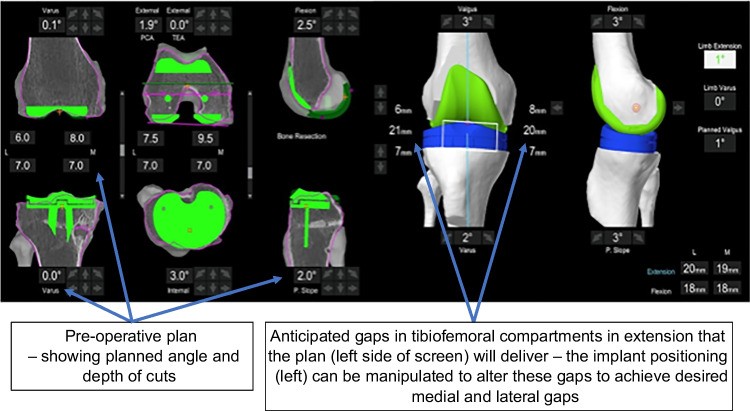
MAKO gap assessment

The OMNIbot in femur-first workflow shows pre- and post-operative laxity by way of curves. Laxity is calculated by subtracting the end coronal range of motion on varus and valgus stress (performed by the senior surgeon) at 0°, 30°, and 90° of flexion (Fig. 1). The MAKO depicts the gaps in the tibiofemoral compartments in two positions (0° and 90°). Laxity is calculated by subtracting the thickness of the implant) from the measured number (Fig. 2).

Imbalances were addressed via recutting the tibia into more varus or valgus or slope as required, up to the functional alignment limits. Any residual imbalance after these limits were reached was addressed with soft tissue release.

### Data collection

Pre-operative patient demographics were collected. Intraoperative data was collected from the operative reports and screenshots taken from the robotic systems. Final resection angles and depths, final limb alignment, releases performed, and implants used were recorded.

### Bone resection

**Fig. 3:**
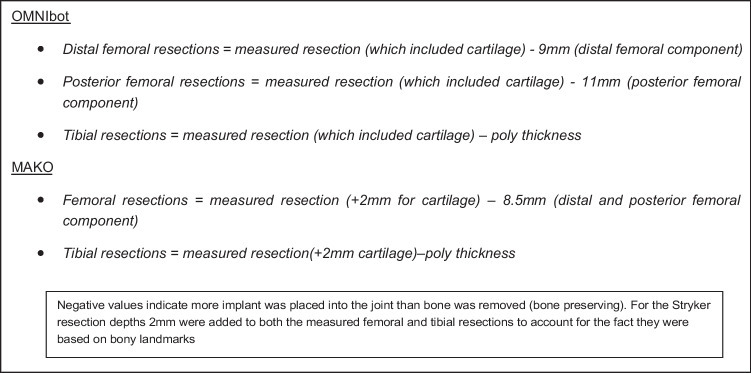
Equations used to determine the amount of bone resected versus implant inserted

To account for differences in the implant designs between the two groups, the amount of bone resected versus implant inserted was determined by the equations in Fig. 3.

### Patient-reported outcomes (PROMS)

Pre-operatively and more than 1 year postoperatively, patients answered the Oxford Knee score (OKS), Veterans Rand 12-item survey (VR-12), and Knee injury and Osteoarthritis Outcome score (KOOS).

### Statistical analysis

All statistical analyses were performed on an intent-to-treat basis. Continuous parametric data was analysed using unpaired Student’s *t*-tests and chi-square tests for categorical data. Significance was set at *p*>0.05 for all tests. Statistical analysis was performed using SPSS (v26).

## Results

**Fig. 4:**
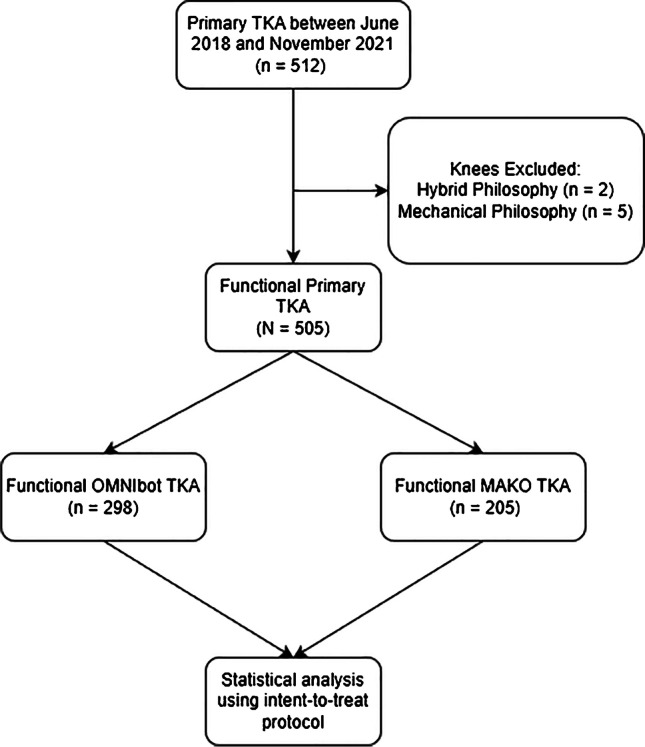
Patient selection

A total of 505 functionally aligned TKAs were performed; 298 (108 bilateral) OMNIbot and 207 (92 bilateral) were performed using the MAKO (Fig. 4).

### Patient demographics

MAKO patients were younger (67 vs 69, p=0.002) than OMNIbot patients (Table 1). There were otherwise no significant differences.

**Table 1:** Pre-operative patient characteristics

	OMNIbot	MAKO	P value
Age, mean ± STD (range)	70 ± 9 (41-91)	67 ± 8 (44-87)	0.001*
Side Left:Right (% right)	145:153 (51%)	89:118 (57%)	0.2
Gender, Male:female (% male)	163:135 (55%)	104:103 (50%)	0.3
Pre-op coronal, mean ± STD (range)	3.2° ± 5.3° varus (18 valgus – 18 varus)	4.1° ± 6.6° varus (15 valgus to 15 varus)	0.06
Pre-op sagittal, mean ± STD (range)	6.6° ± 6.6° flexion (34° flexion – 12° extension)	6.5° ± 8.1° flexion (64 flexion – 10 extension)	0.8
BMI**,** Mean ± STD (range)	31 ± 6 (22–42)	32 ± 9 (20–56)	0.4
Tourniquet, mean ± STD (range)	56 ± 13 min (34 – 120)	61 ± 15 min (42–91)	0.08
Pre-op max Flexion ROM**,** mean ± STD (range)	114° ± 13 (80–135)	110° ± 20 (80–130)	0.2

*p<0.05 compared using independent *t-*tests

### Implant position

#### Angle of cuts

The MAKO femoral components were more externally rotated in relation to the posterior condylar axis (2.3° vs 0.1°, *p*<0.001) and were less valgus (1.6° vs 2.7° valgus, *p<*0.001) than OMNIbot femoral components. There were no differences in external rotation in relation to the TEA (0.3° vs 0.3°, p=0.9) or femoral component flexion (2.2° vs 2.1°, *p=*0.6) between the two groups.

MAKO tibial components were more varus (2.4° vs 1.9° varus, *p*<0.001) and had less of a tibial slope (3.4° vs 3.6°, *p*=0.02) than OMNIbot tibial components (Table 2).

**Table 2:** Angles of intra-operative cuts

	OMNIbot	MAKO	P value
Femur coronal cut, mean ± STD (range)	2.7° ± 1.5 valgus (6 valgus - 2 varus)	1.6°± 1.7 valgus (6 valgus - 3 varus)	<0.001*
Femur Sagittal cut, mean ± STD (range)	2.2° ± 1.6 flexion (5 flexion – 2 extension)	2.1°± 1.7 Flexion (6 flexion – 4 extension)	0.6
Rotation Femur PCA, mean ± STD (range)	0.1° ± 0.9 external rotation (3 external – 2 internal)	2.3°± 2.1 external rotation (7 external - 4 internal)	<0.001*
Rotation Femur TEA, mean ± STD (range)	0.3°± 3.3 external rotation (8 external – 9 internal)	0.3°± 2.2 external rotation (6 external to 5 internal)	0.9
Tibia Coronal cut, mean ± STD (range)	2.1°± 1.1 varus (6 varus – 2 valgus)	2.6°± 1.3 varus (5 varus - 3 valgus)	<0.001*
Tibia Sagittal cut, mean ± STD (range)	3.6°± 1.2 slope (1 – 9)	3.4°± 0.8 slope (1 – 6)	0.02*

All measurements in degrees *p<0.05 compared using independent *t*-tests ** *p*<0.05 compared using chi-square

#### Bone resection

Both systems demonstrate less bone resected than implant inserted on average (were bone preserving).

For the femoral component, OMNIbot TKAs were more bone preserving (compared to the MAKO TKAs. However, only the medial posterior femoral cut had a difference between the systems of greater than 1mm (OMNIbot-0.3mm vs MAKO-1.9mm, *p*<0.001).

**Fig. 5:**
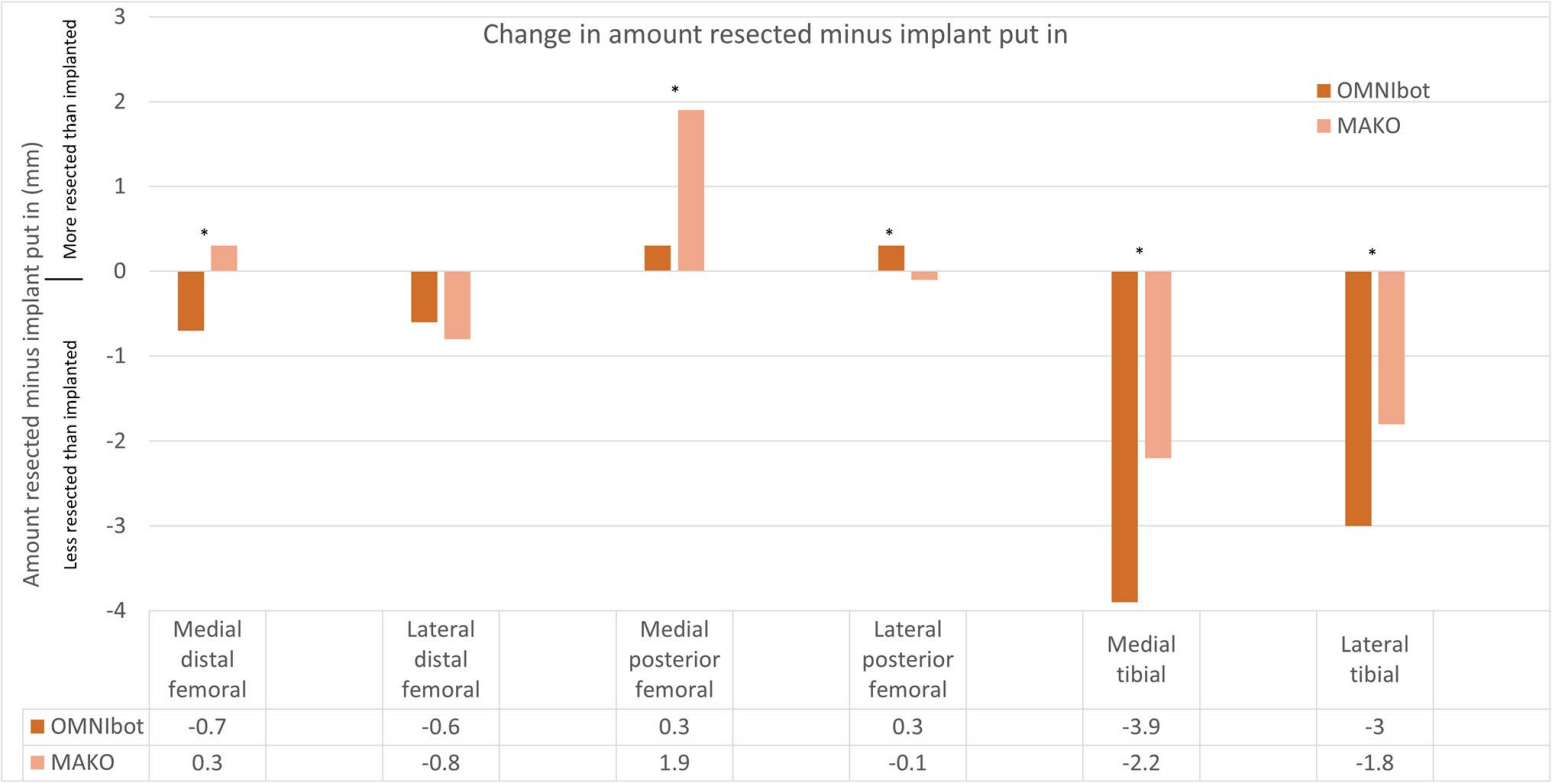
Change in amount resected minus implant put in. Positive values denote more bone was resected than prosthesis put in, while negative values show more implant was put in than bone resected. * *p*<0.001

Regarding the tibia, OMNIbot TKAs were more bone preserving both medially and laterally compared to the MAKO TKAs (medially-3.9mm vs 2.2mm, *p*<0.001, laterally-3mm vs 1.8mm, *p*<0.001) (Fig. 5).

### Post-operative limb alignment and inserts

With MAKO TKA, final HKA was overall more varus (1.1° vs 0.6°, *p*<0.001) and more likely to be outside 3° from the mechanical axis as a group (7% vs 3%, *p*=0.03). Both groups achieved high levels of components in the targeted coronal HKA and tibial coronal range (99% vs 99%, *p*>0.99). The OMNIbot system had a higher rate of components in the targeted femoral coronal range, though both showed excellent rates of achieving the desired range (99.7% vs 98%, *p=*0.04) (Table 3).

**Table 3:** Post-operative limb alignment and patella resection

	OMNIbot	MAKO	P value
Post-op coronal alignment, mean ± STD (range)	0.6°± 1.5 varus (8 valgus to 5 varus)	1.1°± 1.7 varus (4 valgus to 5 varus)	0.01*
Coronal HKA with 3° of mechanical axis, n (%)	(97%)	(93%)	
Post-op sagittal alignment, mean ± STD (range)	0.2 °± 1.4 flexion (7 flexion to 7 hyperextension)	0.4°± 1.3 Flexion (5 flexion to 4 hyperextension)	0.1
Within 5°of full extension, n (%)	296 knees (99%)	201 knees (100%)	0.5
Patella native, mean ± STD (range)	23±3 mm (15-29)	23±3 mm (16-29)	0.3
Minimum Polyethylene, n (%)	149 knees (50%)	118 knees (43%)	0.1
Coronal HKA, n (%) (Within 5° varus to 3° valgus of mechanical axis)	294 knees (99%)	198 knees (99%)	>0.99
Femoral coronal cut, n (%) (Within 6° valgus and 1° varus)	298 knees (99.7%)	200 knees (98%)	0.04*
Max range Tibia coronal cut, n (%) (Within 1° valgus and 5° varus)	293 knees (99%)	203 knees (99%)	>0.99

*p<0.05 compared using independent t-tests

### Recuts and soft tissue releases

OMNIbot TKAs were more likely to have a tibial recut than MAKO TKAs (15% vs 2%, *p*<0.001). There were no differences in femur recut rates or soft tissue releases (Table 4). There was no difference in soft tissue releases when further broken down according to the location and extent of the release (Table 5).

**Table 4:** Intra-operative recuts and soft tissue releases

	OMNIbot	MAKO	P value
Bone recut Femur, n (%)	22 knees (7%)	11 knees (5%)	0.5
Bone recut Tibia, n (%)	46 knees (15%)	5 knees (2%)	<0.001*
Soft tissue release, n (%)	39 knees (13%)	29 knees (14%)	0.8

* *p*<0.05 compared using chi-square test

**Table 5:** Breakdown of soft tissue releases based on location and degree of release

Type of release	Minor (partial/piecrust)	Major release
MCL releases	Number (percentage)	Number (percentage)
OMNIbot	18 knees (6%)	1 knees (0.3%)
MAKO	12 knees (6%)	1 knee (0.5%)
Lateral releases**:** LCL, ITB, popliteus	Number (percentage)	Number (percentage)
OMNIbot	3 knees (1%)	3 knees (1%)
MAKO	2 knees (1%)	7 knees (3%)
PCL	Number (percentage)	Number (percentage)
OMNIbot	6 knees (2%)	3 knees (1%)
MAKO	5 knees (2%)	2 knees (1%)
Anterior**:** retinaculum releases	Number (percentage)	Number (percentage)
OMNIbot	0 knees (0%)	1 knee (0.2%)
MAKO	3 knees (2%)	0 knees (0%)
Posterior**:** Fabella, posterior capsule, meniscofemoral ligament	Number (percentage)	Number (percentage)
OMNIbot	4 knees (1%)	3 knees (1%)
MAKO	3 knees (2%)	0 knee (0%)

## PROMS

A total of 100 MAKO knees with completed OKS-12 scores at more than 12 months post-operatively were age-and side-matched with 100 OMNIbot knees at more than 12 months. There was no difference in cohort demographics other than MAKO patients were generally younger (70 vs 68 *p=*0.03) (Table 6).

**Table 6:** Matching of MAKO and OMNIbot patients for PROM measurements

	OMNIbot (n=100)	MAKO (n=100)	P value
Age, mean ± STD (range)	70±6 (56–86)	68±7 (54–87)	0.03*
Side**,** Left:Right, (% right)	46:54 (54%)	43:57 (57%)	0.8
Gender, Male:female (% male)	60:40 (40%)	49:51 (51%)	0.2
Pre-op coronal, mean ± STD (range)	2.4°± 5 varus (18 valgus – 10 varus)	3.3°± 6 varus (15 valgus – 15 varus)	0.3
Pre-op sagittal, mean ± STD (range)	6°±6 flexion (21 flexion – 10 hyperextension))	6°±7 flexion (27 flexion – 10 hyperextension)	0.8
Pre-op OKS**,** mean ± STD (range)	28± 7 (10–42)	27± 9 (5–46)	0.3
BMI**,** mean ± STD (range)	31±5 (22–42)	32±6 (20–50)	0.7

**Fig. 6:**
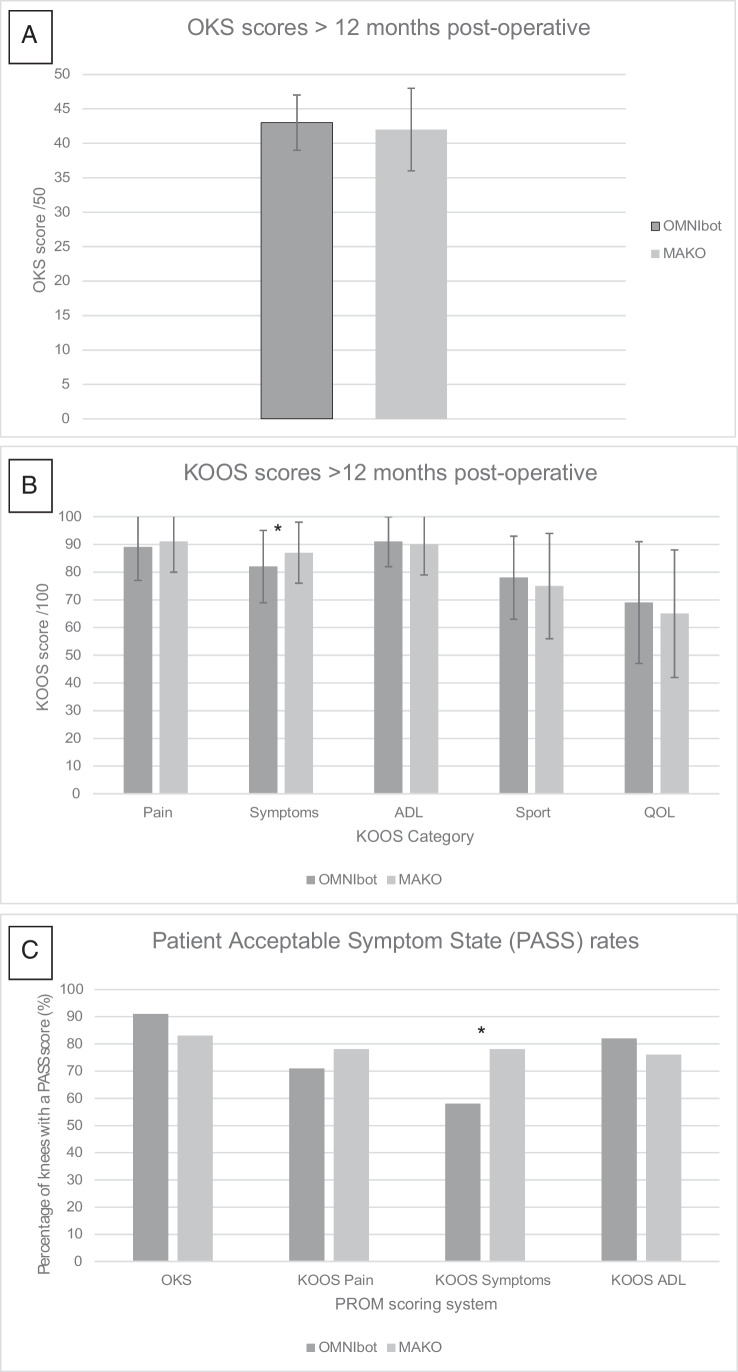
PROM outcomes at more than 1-year post-operative showing **A** average OKS scores (STD), **B** average KOOS subscale scores (STD), and **C** percentage of knees with a patient acceptable symptoms state (PASS)—scores above: OKS >37, KOOS pain >84.5, KOOS symptoms >80.5 and KOOS ADL >83. *p<0.05 [30, 31]

There was no difference in OKS scores or OKS PASS rates (Omni 43 and 91%, and MAKO 42 and 83% respectively) (Fig. 6A/C). MAKO TKAs reported significantly better symptoms according to their KOOS symptoms score than OMNIbot TKAs (87 vs 82, *p*=0.02) (Fig. 6B) and had a higher rate of KOOS symptoms PASS scores (58% vs 78%, *p*=0.02) (Fig. 6C), though while both groups were a minimum of 12 months post-surgery there was a significant difference in follow-up time between the KOOS scores, with MAKOs being collected later (14 vs 20 months, *p<*0.001) (Table 7). Other KOOS subsets showed no differences (Fig. 6B/C).

**Table 7:** Follow-up times and VR-12 scores at minimum 1-year post-TKA [31, 32]

	OMNIbot (n=100)	MAKO (n=100)	P value
Follow-up OKS, mean ± STD (range)	14± 4months (12–26)	15± 6 months (12–36)	0.02*
Follow-up KOOS, mean ± STD (range)	14 ± 5 months (12–36)	20 ± 8 months (12–36)	<0.001*
12-month VR-12 Physical**,** mean ± STD (range)	48± 8 (24–60)	47± 9 (19–59)	0.2
12-month VR-12 Mental**,** mean ± STD (range)	55± 4 (36–64)	54± 10 (20–66)	0.5

*p < 0.05

## Discussion

The improvement in accuracy and consistency of robotically assisted TKA to achieve alignment goals compared to conventional techniques has seen a rapid rise both in their utilisation and number of robotic tools available to choose from. Multiple propriety systems are now available, with little data available currently comparing the effect of different robotic platforms on intra-operative implant positioning, ability to achieve alignment goals, or patient outcomes in TKA. This study compared the effect of using two different robotic tools (MAKO and OMNIbot) and their associated implants (Triathalon and Apex) on implant positioning and short-term patient outcomes, by a single high-volume surgeon using the same alignment and balancing philosophy (functional alignment), and same goals of the surgery.

The most important finding of this study was that using a femur-first measured resection technique with an imageless robotic system (OMNIbot) compared to a quantified total gap balanced technique with the image-based robotic system (MAKO) results in both having a very high rate (99%) of achieving coronal and sagittal alignment goals intra-operatively, are both bone preserving, showed no difference in the rate of soft tissue releases, and achieved high rates of patient satisfaction when used by a single high volume arthroplasty surgeon. These results are achieved with slightly different alignment, different individual component positions, and different workflows depending on the system being used.

TKA performed with the MAKO robot resulted in less valgus femoral and more varus tibial components, more externally rotated femoral components, and slightly more varus limb alignment on average. Equal rates of balanced knees were achieved, with low rates of soft-tissue release and no differences between the groups, though the OMNIbot system required more tibial recuts to achieve balance, due to the workflow of that system. Despite these alignment differences, PROMS at a minimum of 12 months were excellent in both groups. OKS were high in both groups and equivalent (43 in OMNIbot and 42 in MAKO), and while KOOS was higher in the MAKO group, it was at a later time point (20 months vs 14 months),

Whilst the obvious difference between the two platforms is that one is imageless (OMNIbot) and the other is image-based (MAKO), another and perhaps more important difference is the ability to virtually calculate and measure gaps and thus adjust implant position prior to any cuts with the use of the MAKO. This ability allows for a true 6-degree of freedom adjustment of both the femur and/or tibia to achieve balance with the MAKO which is not possible with the OMNIbot in femur-first workflow because the assessment of achieved gaps can only be made AFTER preliminary cuts and trials are made—thus, there are restrictions in how much adjustment of implant position is possible with this approach (only the tibia can be easily recut other than distal femoral resection depth). This explains why the MAKO required less tibial re-cuts, reflecting the advantage of having a system that provides virtual gap information based on the surgeon’s starting plan and allows imbalance to be corrected before the bony cut is made. The same adjustment to the tibal slope is made to balance the knee, but with the MAKO it is done in the virtual environment prior to any bone cuts, while with OMNIbot is done in the real world after the initial cuts have been performed. Interestingly, despite this advantage, OMNIbot surgical times were on average five min faster than MAKO TKA (56 vs 61 min of tourniquet time) perhaps reflecting things like the ergonomics of using the bulkier MAKO device.

Debate remains regarding appropriate gap targets. Traditionally, balancing has been defined as equal tension in both compartments [33]. However wide variations in laxity have been demonstrated in non-arthritic knees [16]. Furthermore, it has been observed for varus-aligned knees, there is greater laxity in the lateral than the medial compartment, and more laxity in flexion than extension [16], although some more recent studies suggest that laxity in flexion may be equal to that in extension [9]. Whilst these subtle variations exist in the literature to defining what is a well-balanced knee, many authors accept some asymmetry between the medial and lateral compartments [9–16, 34, 35]. It is unclear if this should be replicated in prosthetic knees; however, the current study suggests that aiming for equal gaps in extension and flexion on the medial side whilst allowing for either symmetrical or a slight lateral laxity in flexion, achieves excellent clinal outcomes, and can be done with different individual component positing as described. While the true optimal balance targets remain focal points of ongoing research, one of the other benefits of all the navigation and robotic platforms is that they allow for increased quantitative data collection that will help future researchers answer this question as datasets increase in size. With the multifactorial nature of patient satisfaction in TKR, it is likely that “big data” analysis will be the tool for answering this question moving forward.

Despite the improved accuracy and consistency of robotically assisted TKA to achieve alignment goals compared to conventional techniques [36], there is currently limited data demonstrating differences in outcomes between systems or philosophies, with medium to long-term data still to come [26, 37]. This is the first study to compare PROMS between two different robotics TKA systems. We found that the PROMS scores were similar between the two systems, with the OMNIbot TKAs reporting slightly higher OKS scores (43 vs 42, *p*=0.7) while MAKO TKAs reported slightly, higher KOOS scores, with the only significant difference being the KOOS symptoms at more than one year post-operatively (87 vs 82, p=0.02) and the associated PASS rate on the KOOS score. The KOOS difference in symptoms may be due to the difference in follow-up time between the KOOS scores, with the MAKOs being collected later for the KOOS questionnaire (14 vs 20 months, p<0.001), and TKA symptoms having been shown to improve over time [31, 32]. It should also be noted that none of the differences came close to the minimal clinical important difference (MCID) for KOOS or OKS scores indicating that there was no clinically significant difference in short-term outcomes between the groups [38, 39], and excellent scores in both. Studies analysing longer-term data may tease out if there is a functional difference between these two robotic systems.

This study has limitations. Firstly, this study compares not just robotic systems, but also implants (Triathalon and Apex). Whilst the same philosophy and constraint was used (CR) in all cases, the implants differ slightly in geometry and some differences in achieved kinematics and patient outcomes may be due to prosthesis design features. However, this comparison reflects clinical practice, as most robotic platforms are “closed” platforms and only allow for their use with a single implant. Furthermore, the registry reports very good implant survival with the use of both prostheses [40]. There remains a degree of subjectivity in assessing balance, even with increasingly quantitative systems, as the force used for ligament stressing is surgeon dependent and will have a degree of variation. Finally, results were obtained from a high-volume single surgeon, and the results may not be applicable to lower volume surgeons, or those practising a different technique or alignment philosophy to that described.

## Conclusion

This is the first study to directly compare robotic TKAs using either a quantified gap-balancing technique (MAKO) or a measured resection technique (OMNIbot) which shows that while the two techniques and systems result in different implant positions and rates of recuts, both systems achieve equal coronal and sagittal deformity correction and achieve good patient outcomes at short term follow-up
